# Tracing N_2_O formation in full-scale wastewater treatment with natural abundance isotopes indicates control by organic substrate and process settings

**DOI:** 10.1016/j.wroa.2022.100130

**Published:** 2022-02-28

**Authors:** Wenzel Gruber, Paul M. Magyar, Ivan Mitrovic, Kerstin Zeyer, Michael Vogel, Luzia von Känel, Lucien Biolley, Roland A. Werner, Eberhard Morgenroth, Moritz F. Lehmann, Daniel Braun, Adriano Joss, Joachim Mohn

**Affiliations:** a Eawag, Swiss Federal Institute for Aquatic Science and Technology, 8600 Dübendorf, Switzerland; b Department of Environmental Sciences, Aquatic and Isotope Biogeochemistry, University of Basel, Basel 4056, Switzerland; c Laboratory for Air Pollution / Environmental Technology, Empa, Dübendorf 8600, Switzerland; d Department of Civil, Environmental and Geomatic Engineering, ETH, Zürich 8093, Switzerland; e Department of Environmental Systems Science, ETH, Zürich 8092, Switzerland

**Keywords:** Nitrification, Denitrification, Stable isotopes, Isotopomer analysis, Nitrous oxide, GHG mitigation

## Abstract

Nitrous oxide (N_2_O) dominates greenhouse gas emissions in wastewater treatment plants (WWTPs). Formation of N_2_O occurs during biological nitrogen removal, involves multiple microbial pathways, and is typically very dynamic. Consequently, N_2_O mitigation strategies require an improved understanding of nitrogen transformation pathways and their modulating controls. Analyses of the nitrogen (N) and oxygen (O) isotopic composition of N_2_O and its substrates at natural abundance have been shown to provide valuable information on formation and reduction pathways in laboratory settings, but have rarely been applied to full-scale WWTPs.

Here we show that N-species isotope ratio measurements at natural abundance level, combined with long-term N_2_O monitoring, allow identification of the N_2_O production pathways in a full-scale plug-flow WWTP (Hofen, Switzerland). Heterotrophic denitrification appears as the main N_2_O production pathway under all tested process conditions (0–2 mgO_2_/l, high and low loading conditions), while nitrifier denitrification was less important, and more variable. N_2_O production by hydroxylamine oxidation was not observed. Fractional N_2_O elimination by reduction to dinitrogen (N_2_) during anoxic conditions was clearly indicated by a concomitant increase in site preference, δ^18^O(N_2_O) and δ^15^N(N_2_O). N_2_O reduction increased with decreasing availability of dissolved inorganic N and organic substrates, which represents the link between diurnal N_2_O emission dynamics and organic substrate fluctuations. Consequently, dosing ammonium-rich reject water under low-organic-substrate conditions is unfavorable, as it is very likely to cause high net N_2_O emissions.

Our results demonstrate that monitoring of the N_2_O isotopic composition holds a high potential to disentangle N_2_O formation mechanisms in engineered systems, such as full-scale WWTP. Our study serves as a starting point for advanced campaigns in the future combining isotopic technologies in WWTP with complementary approaches, such as mathematical modeling of N_2_O formation or microbial assays to develop efficient N_2_O mitigation strategies.

## Highlights

• ^15^N and ^18^O abundances were applied to trace N_2_O transformations in full-scale WWTP.
• Heterotrophic denitrification was identified as main N_2_O production pathway.
• Seasonal N_2_O emission peaks occurred during winter when NO_2_^−^ accumulates.
• Dosage of reject-water and full aeration of biological treatment increased N_2_O emissions.

## Graphical abstract

**Figure:**
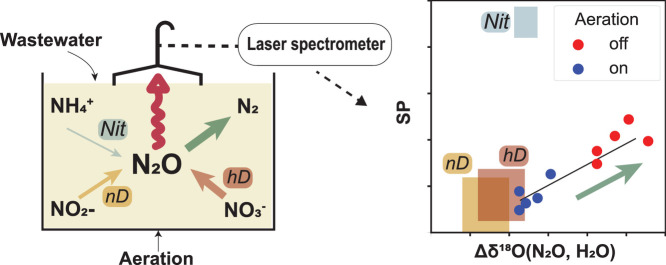

## 1 Introduction

Nitrous oxide is the third most important greenhouse gas and the dominant ozone depleting substance in the stratosphere (IPCC 2013; Ravishankara et al., 2009). Wastewater treatment plants are potent point sources and significant contributors to global anthropogenic N_2_O emissions (Tian et al., 2018; Vasilaki et al., 2019). N_2_O emissions from WWTP exhibit strong temporal dynamics (Gruber et al., 2020). The underlying drivers of these dynamics, however, remain partially unclear, and are likely linked to the complexity of the different nitrogen-cycle reactions involved in N_2_O production in wastewater treatment systems (Domingo-Félez and Smets 2020; Schreiber et al., 2012). Three main metabolic pathways performed by two different groups of bacteria have been identified in WWTPs: (i) hydroxylamine (NH_2_OH) oxidation (Ni) and (ii) nitrifier denitrification (nD) by ammonia oxidizing bacteria (AOB), as well as (iii) heterotrophic denitrification (hD) by heterotrophic denitrifying bacteria (HET) (Ren et al., 2019; Wunderlin et al., 2012). Multiple other microbial and abiotic N_2_O production pathways have been described in literature for specific ecosystems (Butterbach-Bahl et al., 2013) but are not discussed here, to focus on the most relevant processes. However, given a sufficient supply of organic carbon, HET are also able to reduce N_2_O to N_2_, the target product of N elimination in WWTP (Conthe et al., 2018; Pan et al., 2013).

The systematic and efficient mitigation of N_2_O emissions in WWTPs is a challenging task and requires both long-term monitoring of emissions to identify emission peaks, as well as a mechanistic understanding of N_2_O formation mechanisms in the wastewater treatment process (Vasilaki et al., 2019). A number of approaches have been applied successfully in full-scale WWTPs to reduce N_2_O emissions, such as the control of the dissolved oxygen (DO) through different aeration rates and timing (Rodriguez-Caballero et al., 2015; Sun et al., 2017), or different feeding regimes (e.g., step / intermittent feeding) maintaining low *in situ* ammonium concentrations (Hu et al., 2013). However, given the intricacy of N_2_O production and turnover, methods to quantify and to mechanistically understand the pathways involved are essential to explain emission dynamics and develop robust mitigation strategies (Duan et al., 2021).

Differences in stable isotopic substitution of the N_2_O molecule and the bulk isotopic composition of reactive nitrogen substrates ammonium (NH_4_^+^), nitrite (NO_2_^−^), and nitrate (NO_3_^−^), provide valuable information on N_2_O transformation processes, since distinct microbial and/or abiotic pathways exhibit characteristic isotopic signatures (Sutka et al., 2006; Yoshida and Toyoda, 2000). Quantifying the four most abundant N_2_O isotopocules, ^14^N^14^N^16^O, ^14^N^15^N^16^O (^15^N at central, α position), ^15^N^14^N^16^O (^15^N at terminal, β position), and ^14^N^14^N^18^O (Toyoda and Yoshida, 1999) provides three distinct constraints: the bulk ^15^N/^14^N (δ^15^N^bulk^) and the ^18^O/^16^O (δ^18^O) isotope composition as well as the ^15^N site preference (SP). The N and O isotopic compositions of N_2_O are controlled by (1) the composition of the substrate, (2) kinetic isotope effects that occur during N_2_O formation, and (3) kinetic isotope effects associated with N_2_O reduction to N_2_ (Denk et al., 2017; Toyoda et al., 2017; Yu et al., 2020). In addition, the O isotope ratio in the N_2_O pool is influenced by O-atom exchange reactions between water and N intermediate molecules, especially NO_2_^−^ (Casciotti et al., 2002; Lewicka-Szczebak et al., 2016). SP is independent of the substrate isotopic composition and, therefore, an especially sensitive tool for distinguishing mechanisms of N_2_O production and consumption. A powerful way to use the isotopic composition of N_2_O to constrain its formation and processing is the *dual isotope mapping approach*, where SP values are plotted against either Δδ^15^N^bulk^(N_2_O, substrate) or Δδ^18^O(N_2_O, H_2_O) and compared to the isotope signatures known for a given process (Yu et al., 2020). Despite the potential that natural abundance N_2_O isotope measurements offer for pathway characterization, past applications have been almost exclusively limited to laboratory scale reactors (Wunderlin et al., 2013, Tumendelger et al. (2016)).

In this study, we tested, for the first time, whether natural abundance stable isotope measurements in a full-scale WWTP can be applied to characterize N_2_O production pathways under changing inflow composition and process operation. In particular, we evaluated the influence of organic substrate availability and aeration strategies on the N_2_O formation pathways. To further support the estimated contributions of different production pathways and N_2_O reduction, we used measurements of the ^15^N/^14^N and ^18^O/^16^O isotope ratios of N substrates, NH_4_^+^, NO_3_^−^, and NO_2_^−^. Additionally, we performed both spatially and temporally resolved process monitoring of N_2_O emissions and aqueous nitrogen species to interpret the process dynamics during the experiments. Finally, we propose reduction strategies based on the observed emission patterns and attributed pathways.

## 2 Materials & methods

### 2.1 Field site

**Fig. 1:**
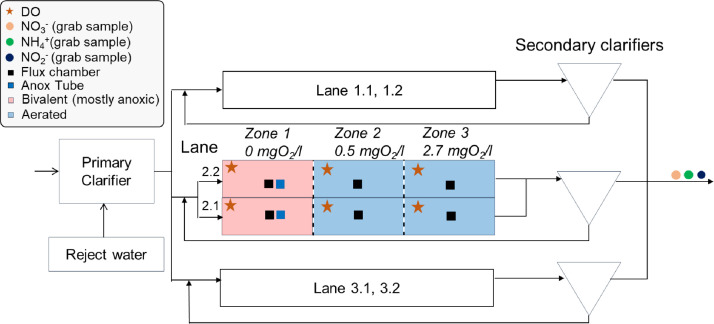
Schematic overview of the Hofen WWTP and installed sensors on lane 2.1 and 2.2 evaluated for this study.

The Hofen WWTP (Switzerland, 47°27′57.3″N 9°23′49.1″E) treats the wastewater of roughly 70,000 population equivalents. After mechanical treatment by screening, grit chambers, and primary clarification, the wastewater enters the biological treatment stage, consisting of six activated-sludge plug-flow reactors, each comprising three cascaded stirred reactors (3 × 530 m^3^, Fig. 1). While organic compounds and N are removed biologically, phosphorus is removed through chemical precipitation using iron(III). This biological treatment scheme represents a standard activated sludge configuration (Tchobanoglous et al., 2014). The average COD and nitrogen load of the treatment plant are 9700 kgCOD/d and 860 kgN/d with average removal rates of 95% and 65%, respectively.

The biological treatment is equipped with multiple liquid-phase sensors for continuous DO (LDO sc, Hach, USA) monitoring (Fig. 1). Effluent concentrations for various nitrogen species (NH_4_^+^, NO_3_^−^, and NO_2_^−^) are measured daily in 24 h composite samples.

The wastewater is evenly distributed over the six treatment lanes. The N removal process is anoxic – oxic, i.e., anaerobic denitrification to N_2_ and aerobic NH_4_^+^ oxidation. The DO concentration is controlled at distinct set-points for each compartment. The first zones are generally operated anoxically and stirred, but can be aerated, as soon as the air consumption in Zone 3 exceeds a defined threshold. This primarily happens during wet weather conditions and in the winter seasons at low temperatures. The second and the third zone are obligatory oxic, i.e. are continuously aerated. Even under aerated conditions, denitrification can proceed within anoxic microsites/microaggregates (Daigger et al., 2007). After the biological treatment to eliminate fixed N, the wastewater enters the secondary clarifiers. Two activated sludge lanes share one secondary clarifier, respectively, and therefore receive the same return sludge (Fig. 1). The biological treatment is operated with a fixed total-solids retention time (SRT) of 13 days. Excess activated sludge is treated in an anaerobic digestion process (not shown in Fig. 1), delivering ammonium-rich reject water to the biological treatment. Increasing the ammonium load in the inflow, reject water is dosed into the primary clarifier to make sure that the N load is equally distributed among the lanes. Typically, reject water from sludge treatment is added overnight from 11 pm to 7 am in batches, every 30 min.

### 2.2 . Continuous N_2_O monitoring

Continuous N_2_O emission monitoring was done using the flux chamber approach, as described in Gruber et al., (2020). A part of the monitoring results (November 2019 – December 2020) has already been presented by Gruber et al. (2021). Flux chambers were installed in Zone 1, 2 and 3 according to Fig. 1. Additionally, 1.5-meter-long columns, called anox tubes (Fig. S.1), were installed in Zone 1 of selected lanes (1.1, 2.1, 2.2, 3.2) to sample N_2_O from the mixed liquor during non-aerated operation by gas stripping with a blower. This technique provides qualitative information on temporal fluctuations of dissolved N_2_O concentrations for Zone 1. N_2_O concentrations from the anox tubes are not quantitative, since the efficiency of the stripping process can only be roughly quantified (Fig. S.2). However, anox tubes provide a temporal trend of dissolved N_2_O concentrations, relevant for interpretation of N_2_O production/consumption processes. A small share of the off-gas from the chambers and anox tubes was diverted to a central N_2_O measuring unit, consisting of an automated valve system, preceding a dehumidifier and a non-dispersive infra-red sensor (X-stream, Emerson, St. Louis MO, USA). The N_2_O monitoring system was installed in October 2019, and since then is running continuously.

### 2.3 . Campaigns with isotope measurements

In 2019, 2020, and 2021 three intensive sampling campaigns supported by N_2_O isotopic measurements were performed on two selected lanes (2.1 and 2.2, Table 1). Campaigns were conducted on days with rather dry weather conditions on the day of sampling, since rain weather reduces emissions substantially (Gruber et al., 2020). Gaseous and aqueous samples of specific zones were collected for isotopic analyses and concentrations measurements during the experiments. Details on the experiments are given in Table 1.

**Table 1:** Dates, experimental details (aeration of Zone 1), gaseous and aqueous samples taken, and research foci for the three campaigns conducted at the Hofen WWTP.

Campaign	Weather conditions	Date	Experiment	Sampling of gas and liquid phase for isotope analysis in zones	Research focus (results section)
1	Short and light rain before and after the experiment	28.11.2019 (09:00–12:00)	Lane 2.1, Zone 1: aerated Lane 2.2, Zone 1: not aerated	Lane 2.1: 1 per Zone 1–3 Lane 2.2: 1 per Zone 1–3 = 6 samples	Impact of process control (Zone 1 aeration) on N_2_O emissions and processes (3.4)
2	Dry weather	08.12.2020 (13:00–15:00)	Lane 2.1, Zone 1: not aerated Lane 2.2, Zone 1: not aerated	Lane 2.1: 1 per Zone 1–3 Lane 2.2: 1 per Zone 1–3 = 6 samples	Identify N_2_O production processes under standard operation (3.2)
3	Dry weather	24.02.2021 (6:00–15:30)	Lane 2.1, Zone 1: not aerated Lane 2.2, Zone 1: aerated	Lane 2.1: Temporal profile, 5 samples in Zone 1–2 = 10 samples	Impact of daily COD and N inflow variation on N_2_O production processes (3.3)

### 2.4 . Collection of gaseous and aqueous samples and isotopic analyses

Gas samples for N_2_O isotopocule analyses were collected from the sampling lines of the N_2_O monitoring system. For this, the respective line was disconnected from the automated multiport inlet system (Gruber et al., 2020) of the off-gas monitoring device, and the sample gas was extracted with a membrane pump (model PM25032-022, KNF Neuberger AG, Switzerland). Gas samples were integrated over 15 to 20 min to ensure representativeness, dehumidified by permeation drying (model PD-50T-72MSS, Perma Pure LLC, USA) and stored in 40 L aluminum coated gas bags (model GSB-P/44, Wohlgroth AG, Switzerland) until analysis at the Laboratory for Air Pollution / Environmental Technology, Empa. For every gas sample a duplicate was collected to check integrity during transport and prevent sample loss; duplicate samples agreed within 0.5 ppm N_2_O for all gas bags.

The abundances of N and O stable isotopes in aqueous or gaseous samples were reported relative to a standard in the δ-notation in per mil (‰) (Werner and Brand, 2001):

(1) $$\delta X\left({\%}_{0}\right)=\;\frac{\left(R_{sample}\;-\;R_{standard}\right)}{R_{standard}}$$

where X refers to the rare isotopocule, i.e. ^15^N and ^18^O for dissolved nitrogen species as well as water and ^14^N^14^N^18^O (abbreviated as ^18^O), ^14^N^15^N^16^O (^15^N^α^) and ^15^N^14^N^16^O (^15^N^β^) for N_2_O, and R_sample_ and R_standard_ are the ratios of the abundance of the least and the most abundant isotopic species in the sample and the standard, respectively. The international scales for nitrogen and oxygen isotope ratios are atmospheric N_2_ (AIR-N_2_) and Vienna Standard Mean Ocean Water (VSMOW) (Mohn et al., 2016; Toyoda and Yoshida, 1999). The average ^15^N composition of N_2_O is referred to as δ^15^N^bulk^(N_2_O) (δ^15^N^bulk^(N_2_O) ≡ (δ^15^N^α^ + δ^15^N^β^)/2) and the difference between δ^15^N^α^ and δ^15^N^β^ is termed the site preference (SP ≡ δ^15^N^α^ – δ^15^N^β^).

For the analysis of δ^15^N and δ^18^O in the dissolved N species (NO_3_^−^, NO_2_^−^, NH_4_^+^), mixed liquor samples from the wastewater reactors collected in parallel with gas samples, were filtered with 0.45 and 0.2 µm single-use membrane filters, and stored refrigerated until further processing (Magyar et al., 2021). Nitrogen and oxygen isotope analyses of NO_3_^−^, NO_2_^−^, and NH_4_^+^ were conducted at the Department of Environmental Sciences, University of Basel, Switzerland. δ^18^O and δ^2^H in wastewater were measured at the Stable Isotope Laboratory of the Department of Environmental System Sciences, ETH Zurich.

#### 2.4.1 N_2_O isotope measurement (gas phase)

N_2_O sample gas concentrations were determined with a non-dispersive infrared spectrometer (X-stream, Emerson, St. Louis MO, USA). Thereafter, sample gases were diluted to ambient N_2_O concentrations (approx. 330 ppb) with high-purity synthetic air using mass flow controllers (Vögtlin Instruments GmbH, Switzerland), and the dilution ratio adjusted after CRDS analysis (G5131-i, Picarro Inc., USA). The isotopocule abundances in the samples were measured using quantum cascade laser absorption spectroscopy (QCLAS), preceded by preconcentration (TREX), as described in Ibraim et al. (2018). All samples were analysed in triplicate and standard deviations for repeated analyses was around 0.5 ‰ for all delta values. For calibration a two-point delta calibration approach was implemented (CG1: δ^15^N^α^ = 2.06 ± 0.05 ‰, δ^15^N^β^ = 1.98 ± 0.20 ‰, δ^18^O = 36.12 ± 0.32 ‰; CG2: δ^15^N^α^ = -82.14 ± 0.49 ‰, δ^15^N^β^ = -78.02 ± 0.52 ‰, δ^18^O = 21.64 ± 0.12 ‰), and instrumental drift, as well as differences in N_2_O concentration corrected (Harris et al., 2020).

#### 2.4.2 Isotope analysis in dissolved N species

The N and O isotopic abundances in NO_2_^−^ were determined using the azide method, where NO_2_^−^ is chemically converted to gaseous N_2_O at low pH (4 to 4.5) (Magyar et al., 2021; McIlvin and Altabet, 2005). For the conversion, a sample volume equivalent to 40 or 10 nmol of NO_2_^−^ (depending on the concentration in the sample) was added to 3 ml of nitrite-free seawater in a 20 ml headspace vial, and crimp-sealed. The seawater is used to maximize N_2_O yield and minimize oxygen exchange during the reaction (Granger et al., 2020). Then, 300 µl of acetic acid-sodium azide solution (1:1 mixture of 2 M NaN_3_ with 20% acetic acid) were injected in the vial, and the mixture was shaken. The reaction was stopped using 200 µl 10 M NaOH after at least 30 min. The pre-processing was conducted on the sampling day, and the samples were stored upside-down at room temperature until analysis. The N and O isotopic composition in the concentrated and purified N_2_O samples were measured using a Delta V Plus gas chromatograph isotope ratio mass spectrometer (GC-IRMS, Thermo Scientific, Germany) interfaced with a customized purge-and-trap system and a GC PAL autosampler (CTC, Switzerland), and standardized using the nitrite reference materials N-7373 and N-10,219 (Casciotti et al., 2007) prepared and measured alongside the samples.

The N isotopic composition of NH_4_^+^ was determined using the hypobromite method, where NH_4_^+^ is chemically converted to N_2_O via NO_2_^−^ (Zhang et al., 2007). Briefly, a sample volume equivalent to 40 nmol of NH_4_^+^ was converted to NO_2_^−^by reaction with 0.5 mL of a 50 µM alkaline hypobromite in a 20 ml headspace vial. Then, this NO_2_^−^ sample was converted to N_2_O by reaction with sodium azide, and the N_2_O was analysed as described in the preceding section. In addition to the nitrite standards N-7373 and N-10,219, international ammonium reference materials (IAEA-N1 and USGS26) were prepared, measured alongside the samples and used to standardize the measurements.

The isotopic composition (N, O) of NO_3_^−^ was measured by conversion to N_2_O with the denitrifier method (Casciotti et al., 2002; Sigman et al., 2001). Prior to the NO_3_^−^ isotope analysis, 1 ml of the filtered sample was pre-treated with 40 µl 0.6 M sulfamic acid in 2 ml Eppendorf tubes for NO_2_^−^ removal. The preparation was neutralized by adding 9 µl 2.5 M NaOH after at least 15 min and before the end of the day. Until further processing, the samples were stored at -20 °C. Then, NO_3_^−^ sample equivalent to 20 nmole was converted to N_2_O by a pure culture of denitrifying bacteria (*Pseudomonas chlororaphis* ATCC 13,985) lacking the NosZ enzyme for N_2_O reduction. The N and O isotopic composition in the concentrated and purified N_2_O samples were measured using a Delta V GC-IRMS (Thermo Scientific, Germany) interfaced with a customized purge-and-trap system and a GC PAL autosampler (CTC, Switzerland), and standardized using international nitrate reference materials (IAEA-N3, USGS32, and USGS34) prepared and measured alongside the samples.

#### 2.4.3 H_2_O isotope measurement

In experiment 3, aqueous samples were analyzed for δ^18^O-H_2_O using the high-temperature carbon reduction method. For that purpose, a high-temperature elemental analyzer (TC/EA; Finnigan MAT, Germany) was coupled to a Delta^plus^XP isotope ratio mass spectrometer via a ConFlo III interface (Finnigan MAT, Germany; (Werner et al., 1999)). The TC/EA was additionally equipped with a custom-made Nafion-trap followed by a 4-port valve (Werner, 2003) between the carbon reduction tube and the GC column. The set-up of the carbon reduction tube follows the “MPI-BGC method” described by Gehre et al. (2004). Water was injected automatically with a GC PAL autosampler (CTC, Switzerland) equipped with a 10 μl gas-tight syringe. Preparation for injection of 0.5 μl of water was made with three washing cycles (3 μl) and five pull-ups. All results were normalized to VSMOW and SLAP, assigning consensus values of 0 and 55.5 ‰ for δ^18^O and 0 and 428 ‰ for δ^2^H to VSMOW and SLAP reference waters, respectively (Coplen, 1988).

### 2.5 . Analyses of reactive N-species

Concentrations of cations (NH_4_^+^-N) and anions (NO_2_^−^-N, NO_3_^−^-N) were analyzed using flow injection analysis (Foss, FIAstar flow injection 5000 analyzer, Denmark) and anion chromatography (Methrom 881 compact IC, Switzerland), respectively.

## 3 Results and discussion

### 3.1 . N_2_O emissions at the Hofen WWTP

**Fig. 2:**
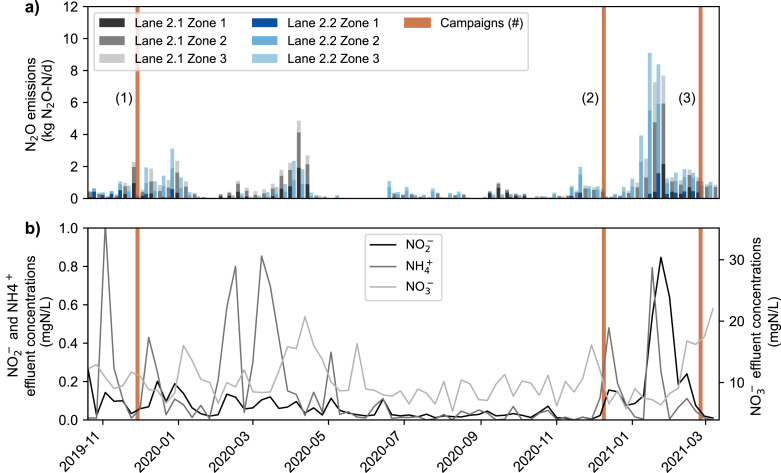
N_2_O emissions of individual zones of lanes 2.1 and 2.2 (panel (a)) and effluent NO_2_^−^, NO_3_^−^ and NH_4_^+^ concentrations of all lanes(panel (b)) at Hofen WWTP. Blue lines indicate the day of the three intensive sampling campaigns and numbers in brackets refer to the campaign number.

The average N_2_O emissions of lane 2.1 and 2.2 at the Hofen WWTP were 0.8 kg N_2_O-N/d during the monitoring campaign (Table 2). The resulting emission factor (0.2% of the total nitrogen load) is low compared to other WWTPs with full-year nitrification and denitrification (median: of 0.4%) (Gruber et al., 2021). Emissions from both lanes displayed similar temporal patterns, with high emissions in winter, and lower emissions during the summer season (Fig. 2). However, the emission pattern is not reproducible in different years. By far the highest N_2_O emissions were observed over several weeks starting in January 2021. The emission peak occurred in parallel with increased NO_2_^−^ concentrations in the effluent of the WWTP, which is known to enhance N_2_O emissions via both nD and hD pathways and has been linked to emission peak phases in other WWTPs (Gruber et al., 2021 b, Ren et al., 2019, Kuokkanen et al., 2021).

**Table 2:** Daily averaged N_2_O emissions on lanes 2.1 and 2.2 for the complete study period, the high emission peak phase, and the single sampling campaigns. Redox conditions in Zone 1, i.e. aeration vs. anoxic, is indicated in brackets.

Phase	Emissions lane 2.1 (kg N_2_O-—N/d)	Emissions lane 2.2 (kg N_2_O-—N/d)
Average (Nov 2019-Mar 2021)	0.8 (standard operation, variable)	0.8 (standard operation, variable)
Peak phase (Jan 2021)	3.6 (aerated)	4.4 (aerated)
Campaign 1	1.9 (aerated)	0.4 (anoxic)
Campaign 2	0.1 (anoxic)	0.3 (anoxic)
Campaign 3	0.7 (anoxic)	1.7 (aerated)

In fact, all lanes were fully aerated during the peak emission phase to increase NO_2_^−^ oxidation capacities of the biological treatment, which in turn favours N_2_O stripping and strongly lowers NO_2_^−^ as well as N_2_O reduction capacities during denitrification. Consequently, during full aeration of Zone 1, emissions in all zones of both lanes increase. However, the major share of the emissions occurs in Zone 2 (Fig. 2), where likely most of the nitrogen turnover happens in case of full aeration of a lane.

The detrimental effect of aeration of Zone 1 (in terms of N_2_O production) compared to anoxic operation was also shown in Campaigns 1 and 3, where the first zone of lane 2.1 or 2.2 were aerated (Table 2). Similarly, in April 2020 only Zone 1 of lane 2.1 was aerated, which led to substantially higher net N_2_O emissions as compared to lane 2.2 (Figs. 2, and 5).

### 3.2 . Identification of N_2_O production pathways using dual isotope mapping approaches

The isotope sampling campaigns at the Hofen WWTP were conducted during different seasons and day times, and under either oxic or anoxic operation of Zone 1 (Tables 1 and 2). The mean SP value for N_2_O emitted from oxygen-replete zones in all three experiment was -1.7 ± 2.7 ‰, which is somewhat lower than results (4.5 ‰) from a previous full-scale WWTP study (Toyoda et al., 2011) and literaure results for N_2_O from Ni, which yields consistently higher SP values (+32.0 to +38.7 ‰). However, values are fully in the range of isotopic signatures measured for nD and hD at a lab-scale WWTP (Wunderlin et al., 2013), as well as in pure culture studies (hD: -7.5 to +3.7 ‰, nD: -13.6 to +1.9 ‰) (summarized in Denk et al. (2017), Ostrom and Ostrom (2017), Yu et al. (2020)). In contrast, N_2_O liberated from Zone 1 under anoxic operation, using the anox tube, displayed significantly higher SP values of 12.3 ± 2.2 ‰. .

**Fig. 3:**
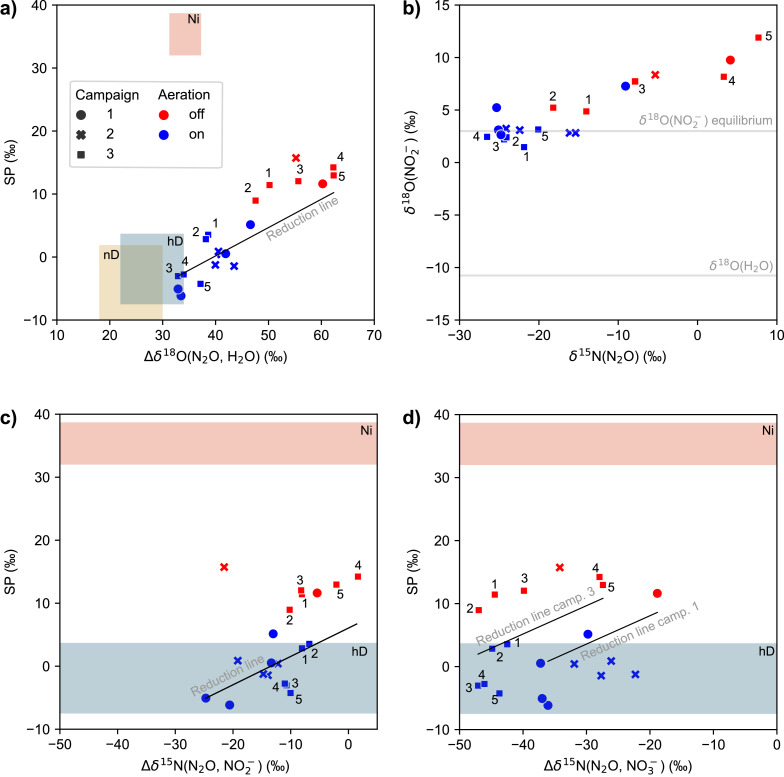
Isotopic signatures of N_2_O liberated from aerated (blue symbols) and anoxic (red symbols) zones of the WWTP Hofen, normalized for the substrate isotopic composition (H_2_O, NO_2_^−^, NO_3_^−^) for the three campaigns that included isotopic measurements. Dual-isotope plots for SP and Δδ^18^O(N₂O, H_2_O) (panel a), Δδ^15^N(N₂O, NO_2_^−^) (panel c), and Δδ^15^N(N₂O, NO_3_^−^) (panel d) are provided. δ^15^N(N_2_O) vs. δ^18^O(NO_2_^−^) values are displayed in panel (b). Gray lines in panel (b) represent the expected δ^18^O values for NO_2_^−^ in equilibrium with water and the measured δ^18^O of H_2_O. Colored areas in panels a, c, and d indicate expected isotopic signatures for N_2_O production pathways (Ni = hydroxylamine oxidation, nD = nitrifier denitrification, hD = heterotrophic denitrification) according to Yu et al. (2020). The expected change in isotopic composition during partial reduction of N_2_O to N_2_ is indicated by black “reduction lines”. For panels (a) and (c), all data points fall on one line, while for panel (c) data points of individual days present individual reduction lines for Campaigns 1 and 3. Numbers next to data points of Campaign 3 (squares) indicate the sampling sequence (t1: 6 – 7 am, t2: 8 – 9 am, t3 = 10 – 11 am, t4 = 1 – 2 pm, t5 = 2:30 – 3:30 pm).

To evaluate the N_2_O production pathways during the experiments in more detail, we applied the dual isotope mapping approach, where SP values are plotted against either Δδ^15^N^bulk^(N_2_O, substrate) or Δδ^18^O(N_2_O, H_2_O) and compared to the isotope signatures known from literature for a given process (Yu et al., 2020). In this approach, the δ^15^N^bulk^(N_2_O) values are corrected for δ^15^N of possible N substrates (NH_4_^+^, NO_2_^-^, NO_3_^−^), with Δδ^15^N^bulk^(N_2_O, substrate) = δ^15^N^bulk^(N_2_O) - δ^15^N_substrate_, while δ^18^O(N_2_O) is compared to δ^18^O(H_2_O), with Δδ^18^O(N_2_O, H_2_O) = δ^18^O(N_2_O) - δ^18^O(H_2_O) (Fig. 3). Wunderlin et al., (2013) followed this approach relating SP to Δδ^15^N^bulk^(N_2_O) values to verify process conditions that are most conducive to distinct production pathways (e.g., hD, nD, Ni) during batch experiments in a laboratory-scale reactor with activated sludge. Since no elevated SP was observed in the aerated zones, no significant contribution of Ni to N_2_O production was anticipated. Moreover, Δδ^15^N(N_2_O, NH_4_^+^) values, which considers ammonium as a possible substrate, did not co-vary with the SP values towards Ni source endmember signatures (Fig. S.3).

Alternatively, Lewicka-Szczebak et al. (2016) showed that a dual isotope mapping approach with SP versus Δδ^18^O(N_2_O, H_2_O) is especially suitable to elucidate mixing of N_2_O produced by hD or Ni and partial N_2_O reduction by denitrification. N_2_O produced by Ni typically bears oxygen isotope values of δ^18^O(N_2_O) ∼ 25 ‰, inherited from atmospheric O_2_ (Frame and Casciotti, 2010). For N₂O produced from hD or nD, the parameter Δδ^18^O(N_2_O, H_2_O) offers additional insights over δ^18^O alone, as discussed below.

The SP values of N_2_O emitted under aerated conditions indicate nD or hD as main N_2_O production pathway. The relationship of SP with Δδ^18^O(N₂O, H_2_O) (Fig. 3a) displays a considerable decrease in both SP and Δδ^18^O(N₂O, H_2_O) during the change from anoxic (Zone 1) to oxic (Zone 2) conditions. This corresponds to a decline in partial N_2_O reduction for Zone 2, in relation to Zone 1, as reduction of N_2_O to N_2_ by hD increases SP of the residual N_2_O pool, since the ^15^N-O bond is more stable than ^14^N-O (summarized in Denk et al. (2017), Ostrom and Ostrom (2017), Yu et al. (2020)). Additional support for the concurrent reduction of nitrite and N_2_O through hD comes from the concomitant increase in δ^18^O(NO_2_^-^) and δ^15^N(N_2_O) shown in Fig. 3b.

Interpreting the Δδ^18^O(N₂O, H_2_O) signatures of N_2_O emitted in the aerobic zone (i.e., in parallel with low SP values) requires a more nuanced interpretation, but yields additional information. The Δδ^18^O(N₂O, H_2_O) value is controlled by both equilibrium isotope effects during O-exchange of precursors with water and branching isotope effects during O-abstraction (Casciotti et al., 2007; Casciotti et al., 2010; Kool et al., 2007). Both effects depend strongly on the bacterial community that performs denitrification, and can differ substantially among systems (Kool et al., 2007; Martin and Casciotti, 2016). The observed δ^18^O(NO_2_^−^) is consistent with complete exchange between NO_2_^−^ and water for samples in the aerated zone; the measured δ^18^O(H_2_O) plus the equilibrium fractionation of 13‰ at 15 to 20 °C yields a composition of ∼3‰ (Buchwald and Casciotti, 2013) (Fig. 3b). Complete exchange can be associated with nitrite produced in nitrification (Buchwald et al., 2012; Casciotti et al., 2010), but can also be mediated by the iron-containing nitrite reductase NirS, which is present in many heterotrophic denitrifiers (Casciotti et al., 2007; Casciotti et al., 2002; Kool et al., 2007). Then, the final Δδ^18^O(N₂O, H_2_O) of N_2_O is determined by the branching kinetic isotope effects associated with nitrite reduction to NO, followed by NO reduction to N_2_O (Casciotti et al., 2007; Casciotti et al., 2002; Martin and Casciotti, 2016; Rohe et al., 2017). The conversion of NO_2_^−^ to N_2_O by the nitrite reductase and nitric oxide reductase enzymes then imparts a branching kinetic isotope effect (Casciotti et al., 2007; Casciotti et al., 2002). The identity of the nitrite reductase enzyme (NirK, NirS) controls the size of this branching isotope effect, and thus δ^18^O(N_2_O, H_2_O). N_2_O production from nitrite that has an equilibrium value of δ^18^O(NO_2_^−^, H_2_O) by bacteria with NirS is associated with a larger oxygen isotope effect and so that N_2_O will display values for Δδ^18^O(N_2_O, H_2_O) of 28 ± 6 ‰, while bacteria with the copper-containing NirK will display a slightly lower Δδ^18^O(N_2_O, H_2_O) of 24 ± 6 ‰ (Martin and Casciotti, 2016). Various hD species are known to have either NirK or NirS, but only NirK has been found in nD (Kozlowski et al., 2016; Nikaido, 2003; Zumft, 1997; Wei et al., 2015). Therefore, N_2_O associated with nD and hD exhibits overlapping ranges for Δδ^18^O(N_2_O, H_2_O), but values greater 30 ‰ are likely to be associated with hD. The only pure-culture constraint on Δδ^18^O(N_2_O, H_2_O) for N_2_O generated by nD, with a value of 22 ‰ (Frame and Casciotti, 2010), falls at the low end of the above-mentioned range, and, thus, consistent with the expectation from the enzyme-based framework provided.

Δδ^18^O(N₂O, H_2_O) values for N_2_O emitted from the aerated zones of WWTP Hofen fall into the range expected for bacteria featuring nitrite reduction using the NirS enzyme (30 to 34 ‰, Fig. 3) and thus a major contribution of hD. This result is also consistent with the observation of Orschler et al. (2021) that although hD can theoretically involve both NirK or NirS, in activated sludge systems, it is predominantly performed via NirS. Δδ^18^O(N₂O, H_2_O) values from the aerated zones are about 10‰ higher than those reported by Lewicka-Szczebak et al. (2016) of 16.7 to 23.3 ‰. The observed discrepancy may be explained by the fact that the underlying values reported by Lewicka-Szczebak et al. (2016) were derived from soil systems that likely differ significantly in terms of the active microbial communities and expressed enzymes, as compared to wastewater systems (Wu et al., 2019).

The prevalence of anaerobic hD under oxic conditions can easily be rationalized by anoxic microsites in sludge flocs even in aerated zones (Sexstone et al., 1985; Daigger et al., 2007). Nevertheless, given the variability seen in Δδ^18^O(N₂O, H_2_O), we cannot exclude a variable contribution from nD under certain conditions, which could be what drives difference between aerobic samples in Fig. 3a. Slightly lower SP and lower Δδ^18^O(N₂O, H_2_O) values may be due to an increased contribution of nD. Alternatively, the higher values may also be caused by a partial reduction of N_2_O also during aerobic phases, assuming that the organic substrate is not fully consumed in Zone 1 and leaks into Zone 2. Furthermore, N_2_O with a high SP and Δδ^18^O(N₂O, H_2_O) might be transported, and mixed in, from Zone 1, as discussed in Section 3.3 in more detail.

Plotting SP values relative to Δδ^15^N(N_2_O, NO_3_^−^) indicates a higher variability among the three intensive sampling campaigns (Fig. 3d). Co-variations in SP and Δδ^15^N(N_2_O, NO_3_^−^) values between N_2_O from aerated and anoxic zones during individual campaigns were driven by the partial N_2_O reduction, indicated by the reduction line. Differences in Δδ^15^N(N_2_O, NO_3_^−^) between experiments, e.g., 31.6 ‰ (Campaigns 1 and 2) versus 41.1 ‰ (Campaign 3), were possibly caused by concentration-dependent variations (affecting cell-specific rates) in the isotope effects associated with denitrification (Kritee et al., 2012). More precisely, the higher NO_3_^−^ concentrations during experiment 3 (10–18 mg NO_3_^−^-N/L) compared to experiment 1 and 2 (0–7 mg NO_3_^−^-N/L) may manifest in substantially higher isotope effects. The increased nitrate concentrations were due to the full aeration of all zones over multiple weeks before experiment 3. The operation led to reduced denitrification activity and NO_3_^−^ accumulation in the biological treatment.

Interestingly, Δδ^15^N(N_2_O, NO_2_^−^) was more consistent than Δδ^15^N(N_2_O, NO_3_^−^) between campaigns, i.e., isotope effects seemed less strongly affected by N substrate concentrations (Fig. 3c). Therefore, isotopic signatures for samples from aerated and anoxic compartments cluster significantly closer to the predicted reduction line (Fig. 3c). The observed correlation of delta values for individual campaigns hence supports the notion that the isotopic composition of NO_3_^-^, NO_2_^−^ and N_2_O are mostly controlled by the sequential reduction of NO_3_^−^ to N_2_ during complete denitrification.

In summary, the isotopic composition of N_2_O, NO_2_^−^, and NO_3_^−^ consistently point towards a high contribution of hD to N_2_O production during aeration on all days. nD may be of variable relevance, yet Ni can be excluded as a significant contributor. hD was previously shown to govern N_2_O production during aeration under low C:N conditions (Domingo-Felez et al., 2016). Our data confirm that obligate anaerobic processes, such as hD, play an important role even during aerated reactor conditions, supported by strong oxygen gradients and anoxic microniches in sludge flocs (Daigger et al., 2007). For zones under anoxic process conditions, observed isotope patterns provide clear evidence for substantial N_2_O reduction. To diagnose the contribution of different production pathways, the relation of SP and Δδ^18^O(N₂O, H_2_O) turned out to be more sensitive than the Δδ^15^N(N_2_O, substrate) approaches. However, combining both approaches as shown here, has the benefit of being able to additionally validate interpretations, and to provide independent process information to assess the full complexity of concurrent N_2_O formation and reduction.

### 3.3 . Diurnal variation in N_2_O emissions and production pathways

**Fig. 4:**
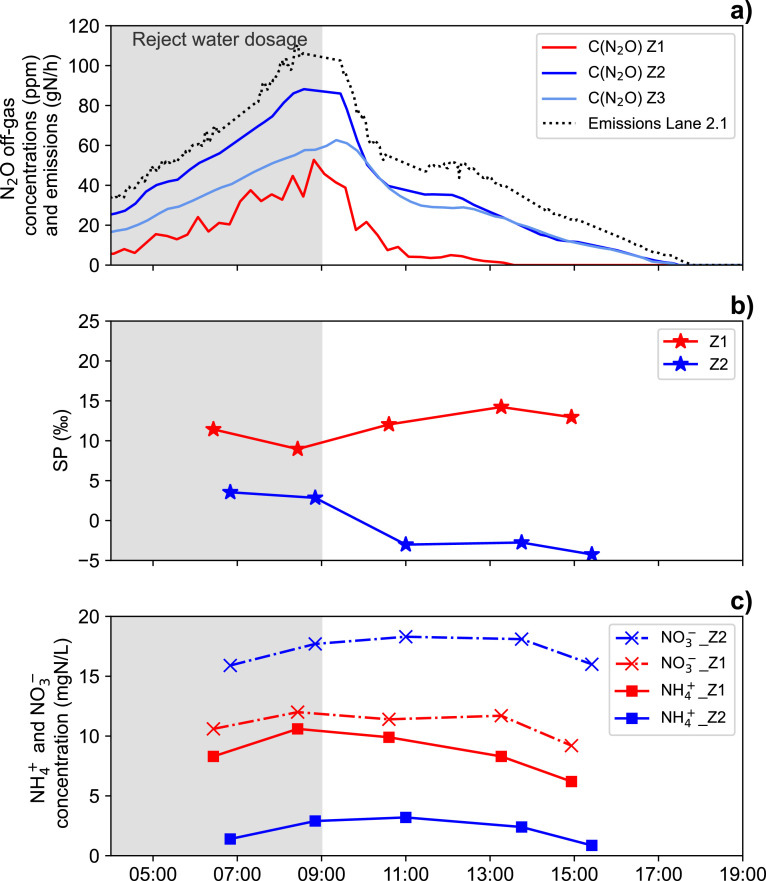
(a) N_2_O concentrations measured in different zones of lane 2.1, and calculated N_2_O emissions. When comparing N_2_O concentrations of Zone 1 to other zones, it needs to be noted that the gas phase in the anox tubes applied in Zone 1 is not in equilibrium with the liquid phase. We anticipate a three times higher concentration under equilibrium conditions (Fig. S.2). (b) N_2_O SP in Zone 1 and 2, indicating a minimum in N_2_O reduction in Zone 1 around 9 am, while N_2_O SP in Zone 2 is generally low but increased at high concentrations in Zone 1 due to transport. (c) NH_4_^+^ and NO_3_^−^ concentrations in Zone 1 and 2 of lane 2.1 are stable despite higher NO_3_^−^ inflow (Fig. S.5), pointing towards high denitrifying activity at 11 am. The gray shaded area shows the period of reject water dosage. The timing of gas and liquid sampling is indicated by markers in Fig. 4b and c: t1: 6 – 7 am, t2: 8 – 9 am, t3 = 10 – 11 am, t4 = 1 – 2 pm, t5 = 2:30 – 3:30 pm.

The main focus of the third campaign was to investigate the effect of the diurnal patterns in N loading (controlled by reject water dosage) and COD substrate inflow on N_2_O emissions and variations in N_2_O reduction. For this, we analysed the isotopic signatures of N_2_O and nitrogenous substrates in Zone 1 and 2 for five different time points during one day at lane 2.1 (Fig. 4). N_2_O emissions exhibited a clear diurnal pattern, with a peak at 9 am, right before the reject water dosage was stopped (Fig. 4a). N_2_O concentration changes in the anoxic zone, measured with the anox tube, were consistent with changes in the N_2_O flux from Zone 2 and 3. While NH_4_^+^ concentrations also exhibited a clear diurnal variation pattern, NO_3_^−^ concentrations were relatively stable throughout the study period (Fig. 4c, Figs. S.4 and S.5, (SI)). NO_2_^−^ was highest in Zone 1 and gradually decreased in Zone 2 and 3, respectively (Fig. S.6 (SI)).

The diurnal trend of the N_2_O site preference in Zone 1 indicates a decreasing importance of N_2_O reduction from 7 am to 9 am (sampling points 1 and 2), also shown in the *dual isotope mapping approach,* e.g., for SP vs. Δδ^18^O(N₂O, H_2_O) (Fig. 3a). After 10 am, SP and Δδ^18^O(N₂O, H_2_O) values for N_2_O from Zone 1 increased along the predicted reduction line, which suggests a return to an increasing relevance of N_2_O reduction for samples 3 to 5. NO_3_^−^ concentrations remain stable in Zone 1 (Fig. 4c) despite an increase of NO_3_^−^ inflow from the return sludge (Fig. S.5 (SI)), confirming that heterotrophic nitrate reduction (hD) was very active after 9 am. We suggest two main causes for the strong daily variation in N_2_O emissions and N removal.

First, the dosage of reject water and the morning peak in N inflow, typically seen in WWTPs, led to a NH_4_^+^ concentration increase (Fig. 4c, t1 – t2), while the N_2_O reduction capacity of the WWTP was lower due to the increased supply of NO_3_^−^. Second, and more importantly, the availability of organic substrate typically exhibits daily fluctuations. Therefore, despite high NH_4_^+^ loads from 10 am to 2 pm (t3 – t4), high availability of organic substrate led to increasing nitrogen removal and, in turn, increased fractional N_2_O reduction rates. Notably, COD concentrations were not measured during the campaign, but are expected to correlate with the inflow rate to the wastewater treatment plant, which exhibits reproducible daily variation (Fig. S.7 (SI)).

The N_2_O SP in Zone 2 is at its maximum between 6 and 9 am, probably due to transport of N_2_O produced in Zone 1, where both N_2_O production and reduction were high during this part of the diurnal cycle, as described above (Fig. 4b). This would imply that N_2_O emissions from Zone 2, before and during the peak phase, i.e., the end of the reject water dosage, comprise a substantial contribution of N_2_O from Zone 1. hD as the main source of this N_2_O is supported by the high Δδ^18^O(N_2_O, H_2_O) values (36.2 ± 2.3 ‰). Alternatively, high SP values in Zone 2 before 9 am can be explained by partial N_2_O reduction, but this is unlikely given COD limitation during reject water dosage. Moreover, transport of N_2_O produced in an anoxic zone to an aerobic zone has been reported earlier for other WWTPs (Mampaey et al., 2016). After 10 am, the difference in SP values between Zone 1 and 2 was increasing again, indicating that N_2_O transport and mixing was less important.

In addition, the contribution of nD to N_2_O formation might have increased after 10 am in Zone 2, which could further explain the lower SP and Δδ^18^O(N₂O, H_2_O) here. Nevertheless, we believe that hD also contributed a major part to the emissions in the aerobic zones between 11 am and 4 pm, given the still-high Δδ^18^O(N_2_O, H_2_O) values.

### 3.4 . N_2_O emissions depend on process operation

**Fig. 5:**
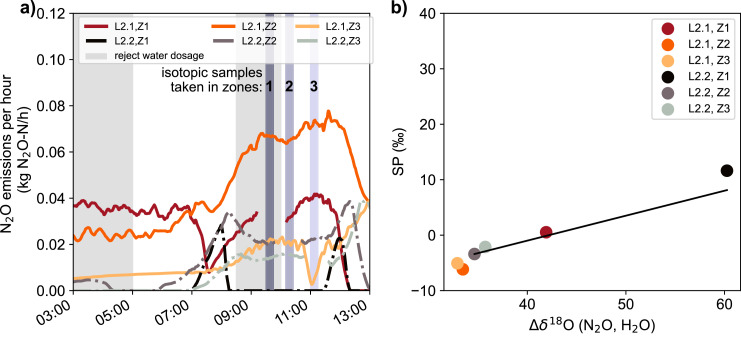
N_2_O emissions during Campaign 1, indicating higher emissions for lane 2.1, where Zone 1 was aerated, as compared to conventional operation in lane 2.2 (Zone 1 anoxic). The vertical lines indicate the timing for isotopic samples. Lane 2.2. Zone 1 was aerated for a short period between 7 and 8 am, and from 11:30 to 12 am, resulting in the increase in N_2_O emissions (panel a). SP and Δδ^18^O(N_2_O, H_2_O) for N_2_O emitted from lanes 2.1 (Zone 1 aerated) and 2.2 (Zone 1 anoxic), indicate a higher share of N_2_O reduction for Zone 1 of lane 2.2, consistent with lower emissions. The indicated straight line represents the expected change in isotopic signatures with progressive N_2_O reduction, the so-called “reduction line” (panel b).

The seasonal dynamics in N_2_O emissions indicate that phases when the air consumption in Zone 3 exceeded a defined threshold, and thus when Zone 1 was aerated, were generally characterized by high net N_2_O production (Fig. 2). To better understand the effect of aerobic conditions in the first zone on overall N_2_O formation, we compared the isotopic signatures of N_2_O produced along a fully aerated lane (2.1) and a lane under standard operation, i.e., with anoxic conditions in the Zone 1 (2.2) (Table 1). The episodes of reject-water dosage in the morning had a high impact on the emissions (i.e., high N_2_O emissions in Campaign 3), but N_2_O emissions were even higher from the fully aerated lane (Table 2). The difference between lanes was primarily driven by emissions in Zones 1 and 2, while emissions in the third zone were comparable (Fig. 5a).

The explanation for increased N_2_O emissions from the fully aerated lane 2.1 can be assessed when comparing isotopic signatures of the N_2_O released from Zone 1 of both lanes (Fig. 5b, Campaign 1). The N_2_O isotopic signature measured in the Zone 1 of lane 2.2, with conventional operation, i.e., Zone 1 mostly anoxic, indicates a substantial reduction of N_2_O. In contrast, for lane 2.1, with Zone 1 aerated, the share of N_2_O reduction was substantially lower. The proportion of N_2_O reduction can be estimated quantitatively by the expression ΔSP = εSP x ln f (Jinuntuya-Nortman et al., 2008; Lewicka-Szczebak et al., 2017; Mariotti et al., 1981), with ε being the enrichment factor (-8.2 to -2.9 ‰, (Yu et al., 2020)), and f the fraction of unreacted N_2_O. The isotopic enrichment factor between product P and substrate S is defined as εX_P/_*_S_* = αX_P/S_ – 1 = δX_P_ / δX_S_ – 1, where α is the isotopic fractionation factor. Applying this approach yields an estimate of 92% of N_2_O (84 to 99% using max and min fractionation factors) reduced for the anoxic Zone 1 of lane 2.2, while only 68% (56 to 90% using max and min fractionation factors) is reduced in the aerated Zone 1 of lane 2.1 (assuming that the SP values for N_2_O from Zone 2 are representative for the N_2_O production process). As during Campaign 3, N_2_O production was very likely driven by hD, given the increased Δδ^18^O(N_2_O, H_2_O) values (35.2 ± 0.6 ‰) in the aerobic zones.

Campaigns 1 and 3 revealed that organic carbon availability, aeration of Zone 1, and reject-water N dosage are the most important modulators of N_2_O emissions during standard operation at the Hofen WWTP, and at a given time of the year. Notably, emissions were lowest in Campaign 2 (Table 2), with anoxic conditions in Zone 1 of both lanes, without reject-water dosage and sampling times in the afternoon, where increased organic substrate concentrations are expected. While it seems relatively clear that aerobic conditions in Zone 1 and low organic substrate availability both lead to higher emissions by impairing a more efficient N_2_O reduction, the mechanism behind the increased production of N_2_O caused by elevated reject-water dosage (which leads to an increase in NH_4_^+^ concentrations) is not fully understood (Gruber et al., 2020). Most plausibly, elevated N_2_O emissions are directly linked to the high NH_4_^+^ concentrations (following substrate- vs- intermediate product systematics). Alternatively, it is possible that the composition of the reject water is somehow unfavorable for heterotrophic denitrifiers and nitrifiers. Further research is needed to unravel underlying mechanisms, e.g., by comparing the effects of dosages of reject-water NH_4_^+^ versus (NH_4_)_2_SO_4_ solution in activated sludge. Nevertheless, our results already yield important information regarding efficient strategies to reduce N_2_O emissions during normal operation at the Hofen WWTP. The guiding principle for the mitigation of N_2_O emissions is to maximize N_2_O reduction by avoiding aeration of Zone 1, and dosing reject-water primarily during periods with high organic carbon load, e.g. in the afternoon. The adaptation of the feeding strategy to optimize organic carbon utilization towards most efficient N_2_O reduction has been successfully applied in side-stream treatment (Peng et al., 2017). However, changing reject-water dosage operation strategies should be critically evaluated, as the effects of the NH_4_^+^ loading are multifaceted. That is, besides potential impacts of the NH_4_^+^ dosage on net N_2_O emissions, other constraints need to be considered. For example, increased NH_4_^+^ peak concentrations can lead to NH_4_^+^ breakthrough, and load equilibration in the diurnal pattern is beneficial for the nitrification performance (Meyer and Wilderer 2004). We propose to apply conventional activated sludge modeling and full-scale testing, combined with extensive process monitoring, to optimize reject-water dosage in terms of effluent quality and maximized reduction capacities for N_2_O mitigation (Henze et al., 2000).

Isotopic technologies were successfully applied to analyze the contribution of N_2_O production pathways at the Hofen WWTP, and provided mechanistic understanding to support mitigation strategies. Still, long-term monitoring of the isotopic composition of N_2_O and other nitrogen species is needed in future studies to evaluate the consistency and robustness of the approach. A major advantage to characterize contributions of N_2_O reduction and production pathways at the Hofen WWTP involved the cascaded lanes, with clearly defined redox conditions in each zone. We expect that the application in flow-through, non-compartmented activated sludge systems can be more challenging due to increased mixing over a whole lane, leading to a higher exchange of the nitrogen pools. Furthermore, continuous long-term monitoring is important for the extrapolation and interpretation of the data and the characterization of the seasonal emission peaks. The lion's share of the total annual N_2_O emissions can be attributed to the January peak emission period (Fig. 2; 50% of the total emissions) in association with elevated NO_2_^−^ concentration levels. Seasonally impaired NO_2_^−^ oxidation in WWTPs, leading to NO_2_^−^ accumulation, has been linked to low abundances of nitrite oxidizing bacteria (NOB) and drastic changes in the whole activated sludge microbial community (Gruber et al., 2021). However, the NOB loss observed by Gruber et al. (2021) at the Uster WWTP led to NO_2_^−^ accumulation over a periods of 1–2 months, and it is unclear whether similar process were also responsible for the accumulation of nitrite over a few weeks at the Hofen WWTP.

## 4 Summary and conclusions

- Measurements of relative ^15^N and ^18^O abundances in nitrogen-bearing molecules were successfully applied to characterize dynamics of N_2_O formation pathways under normal operation in a full-scale activated sludge WWTP. N_2_O was mainly produced by heterotrophic denitrification, while nitrifier denitrification appeared to be less important and of rather variable influence; NH_2_OH oxidation was negligible.
- Seasonal emission peaks occurred during winter when NO_2_^−^ accumulates, and when the biological treatment is operated at full aeration, but NOB activity is still impaired.
- Based on N_2_O isotopic measurements, N_2_O reduction was identified under anoxic conditions, and to lesser extent also under oxic conditions, when it is restricted to anoxic micro-niches. Fractional N_2_O reduction was most pronounced under organic-substrate-replete conditions, while N_2_O accumulation in the anoxic zone was primarily observed when organic substrate was limiting. Hence, the daily variation of organic substrate has a strong impact on the reduction of N_2_O, and in turn, diurnal N_2_O emission fluctuations.
- The dosage of reject-water and full aeration of the biological treatment significantly increased N_2_O emissions, since N_2_O reduction was strongly impeded. Hence, an efficient mitigation strategy towards optimized N_2_O reduction may involve shifting reject-water dosage to periods with high organic substrate availability, as well as avoiding full aeration of the biological treatment.
- Coupling isotopic technologies with continuous long-term monitoring of N_2_O emissions is a powerful tool for qualitative N_2_O pathway identification and the development of N_2_O mitigation strategies in full-scale WWTPs. However, clearly defined conditions in a reactor system are required to interpret the data.

## Declaration of Competing Interest

The authors declare that they have no known competing financial interests or personal relationships that could have appeared to influence the work reported in this paper.

## Appendix Supplementary materials

Image, application 1
