# Supplementary material for: Tracing N2O formation in full-scale wastewater treatment with natural abundance isotopes indicates control by organic substrate and process settings
Source: Water Res X. 2022 Feb 28;15:100130. doi: 10.1016/j.wroa.2022.100130 (PMC8917317; doi:10.1016/j.wroa.2022.100130)
Supplement: Supplementary file 1 [file mmc1.docx]

**Tracing N_2_O formation in full-scale wastewater treatment with natural abundance isotopes indicates control by organic substrate and process settings**

Wenzel Gruber ^a,*^, Paul Magyar ^b,*^, Ivan Mitrovic ^a^, Kerstin Zeyer ^c^, Michael Vogel ^d^, Luzia von Känel ^d^, Lucien Biolley ^d^, Roland A. Werner ^e^, Eberhard Morgenroth ^a^, Moritz F. Lehmann ^b^, Daniel Braun ^d^, Adriano Joss ^a^, Joachim Mohn ^c,o^

^*^ First authors with equal contributions

^o^ Corresponding author

^a^ Department Process Engineering, Eawag, 8600 Dübendorf, Switzerland

^b^ Department of Environmental Sciences, Aquatic and Isotope Biogeochemistry, University of Basel, 4056 Basel, Switzerland

^c^ Laboratory for Air Pollution & Environmental Technology, Empa, 8600 Dübendorf, Switzerland

^d^ Department of Civil, Environmental and Geomatic Engineering, ETH, 8093 Zürich, Switzerland

^e^ Department of Environmental Systems Science, ETH, 8092 Zürich, Switzerland

Submitted to Water Research X on -----.

## Monitoring setup


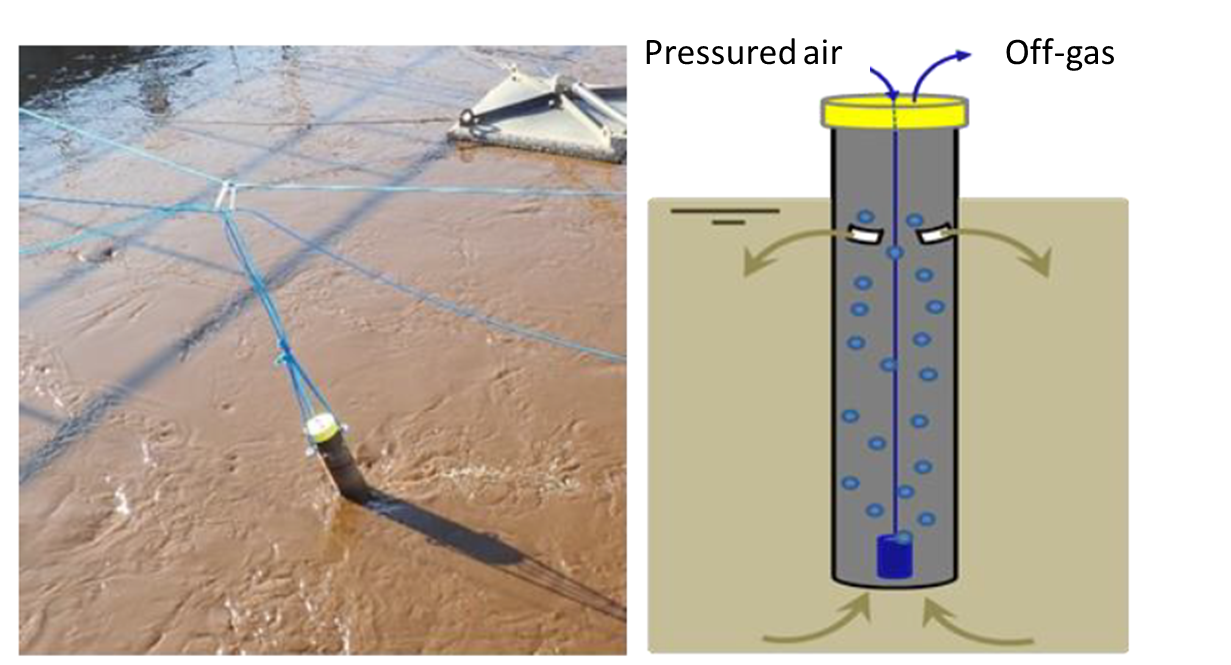


Figure S.1 ‘Anox Tube’ for off-gas stripping from unaerated zones


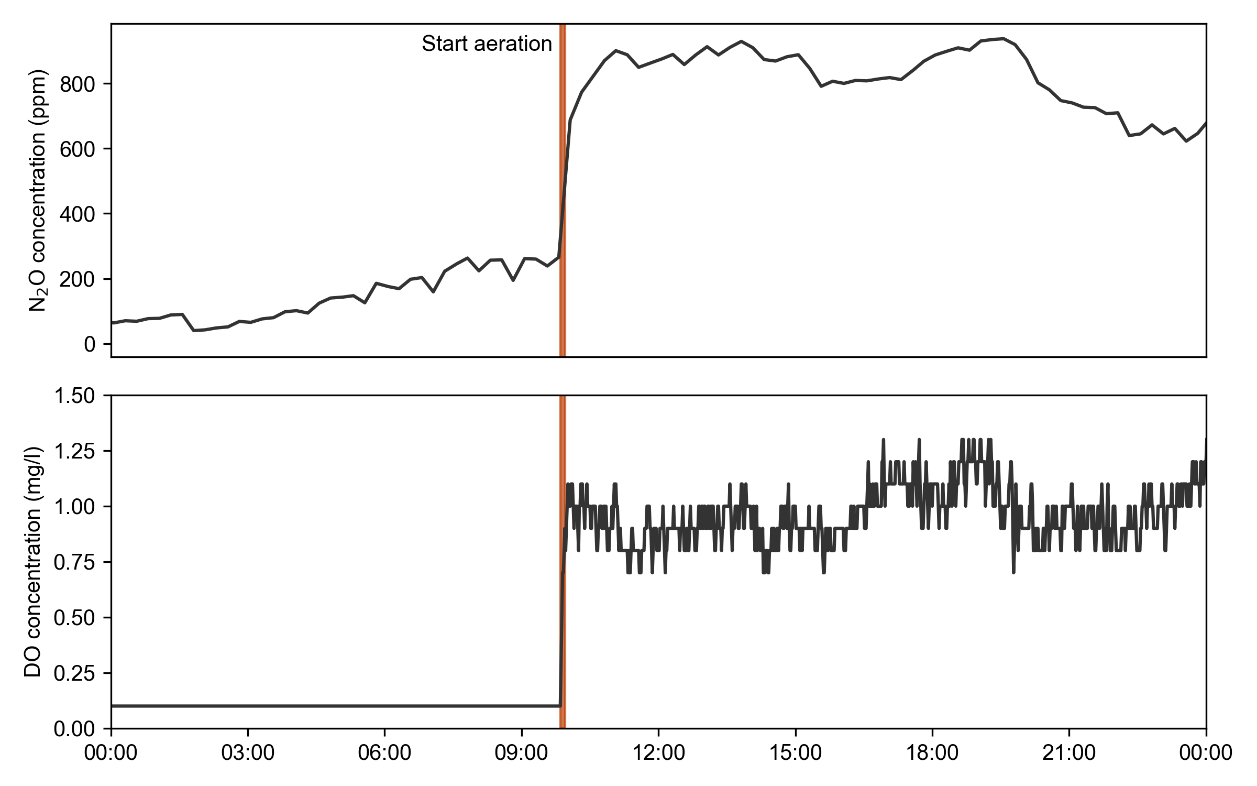


Figure S.2 Measured N_2_O (off-gas) and DO concentration in zone 1 of lane 2.1 before and during aeration. The immediate increase of the N_2_O concentration after the start of the aeration can be mainly linked to increased stripping of N_2_O. The concentration jumps from roughly 300 ppm to 800 ppm. Hence, a stripping efficiency of 30 to 40% is assumed for the anox tube.

## Isotopic measurements


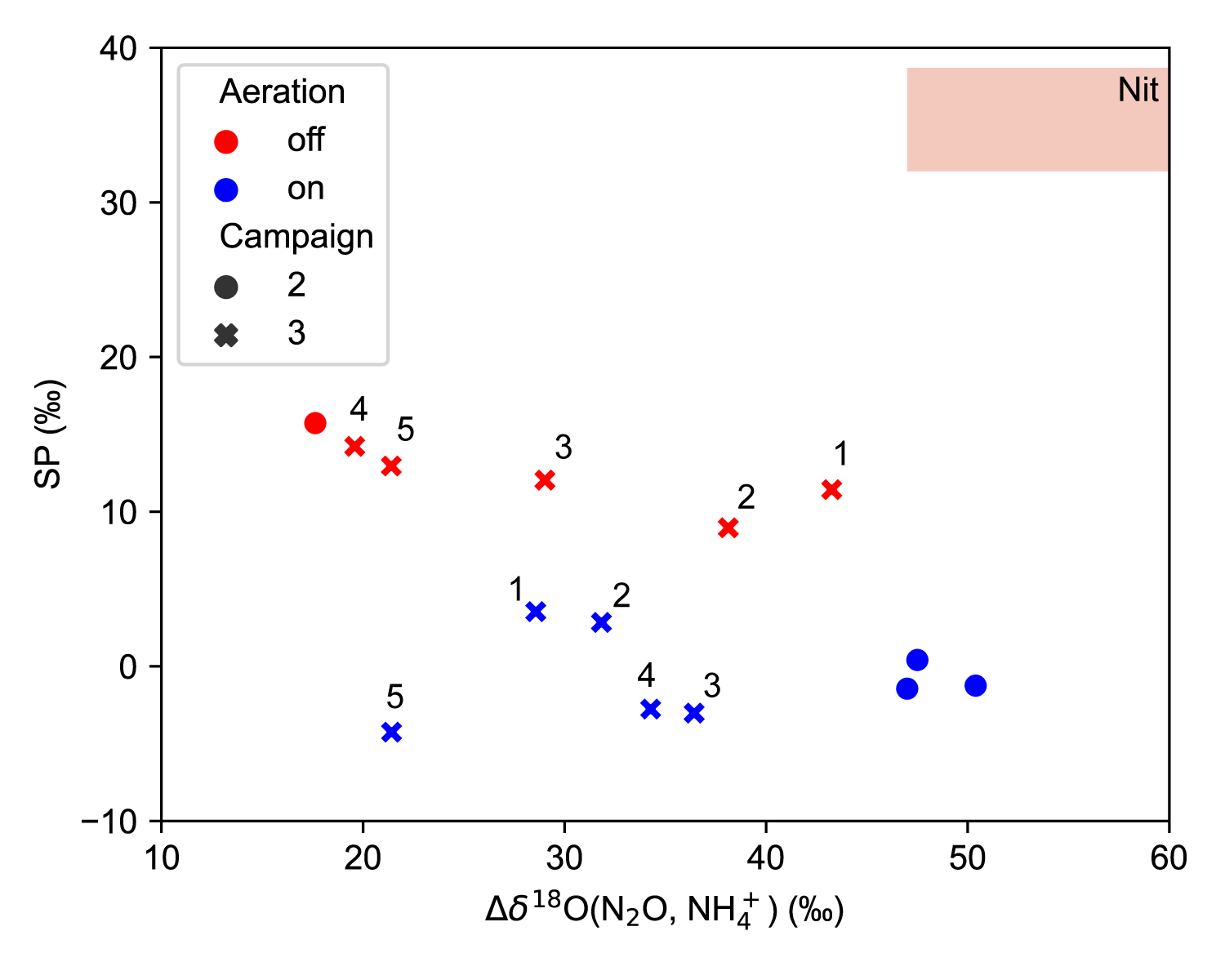


Figure S.3: Relation between Δδ^15^N(N_2_O, NH_4_^+^) and SP in campaign 2 and 3. Numbers next to campaign 3 data points (stars) indicate sampling sequence.


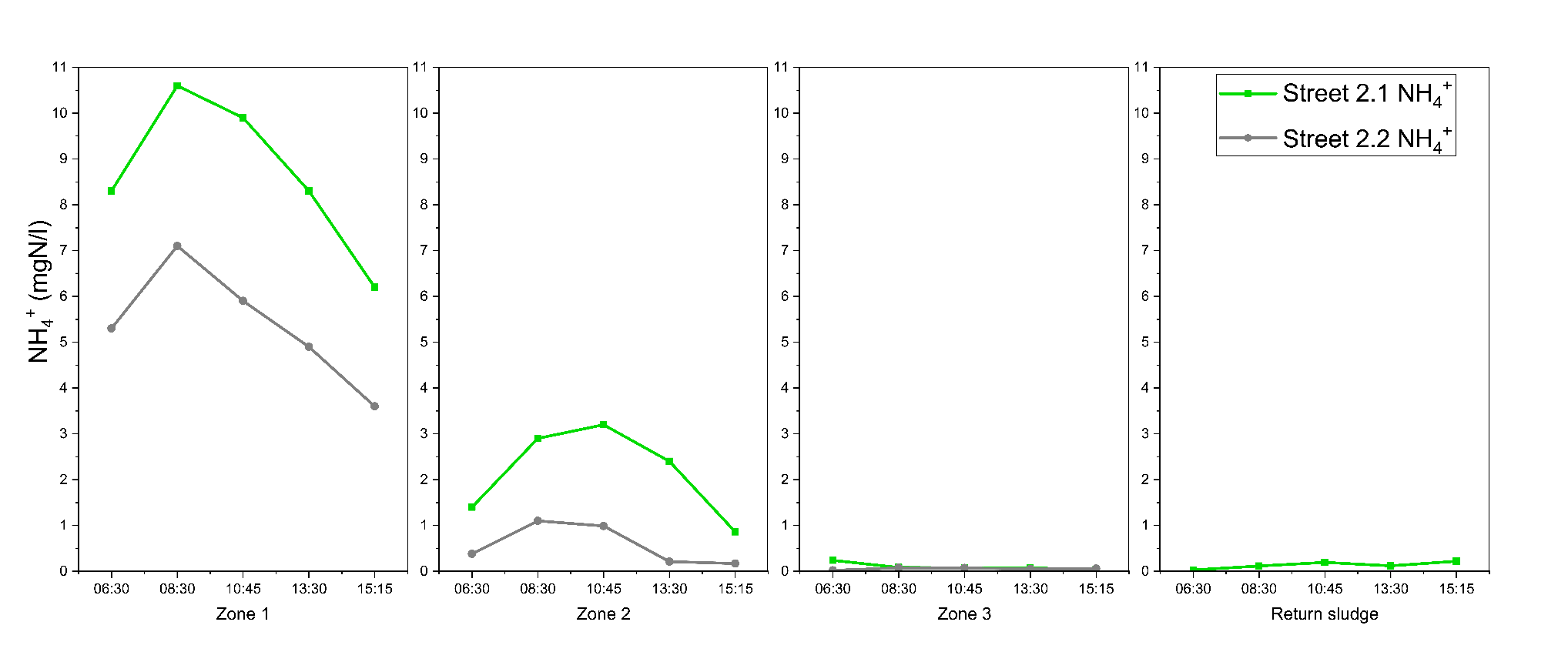


Figure S.4: Measured NH_4_^+^ concentrations during campaign 3 in zones 1, 2, 3 and in the return sludge.

## S3. Monitoring data


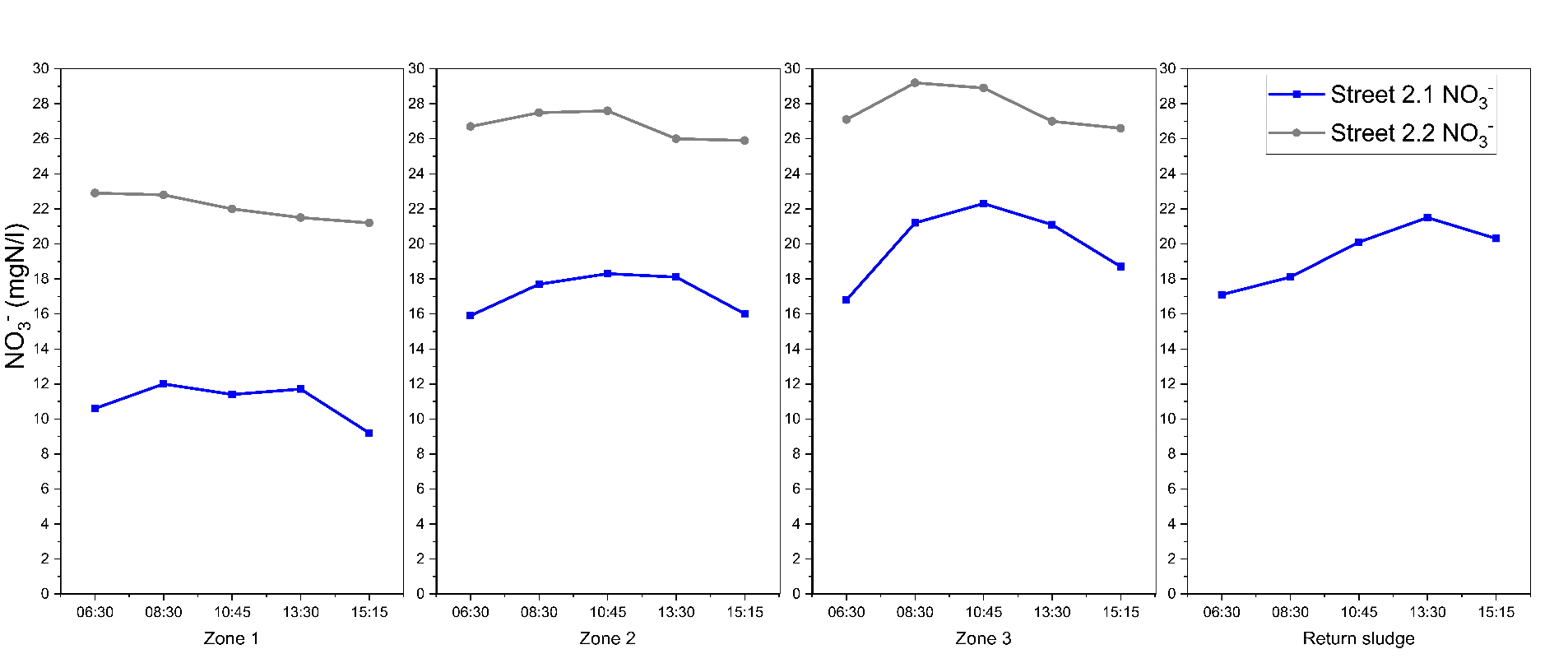


Figure S.5: Measured NO_3_^-^ concentrations during campaign 3 in zones 1, 2, 3 and in the return sludge.

Figure S.6: Measured NO_2_^-^ concentrations during campaign 3 in zones 1, 2, 3 and in the return sludge.


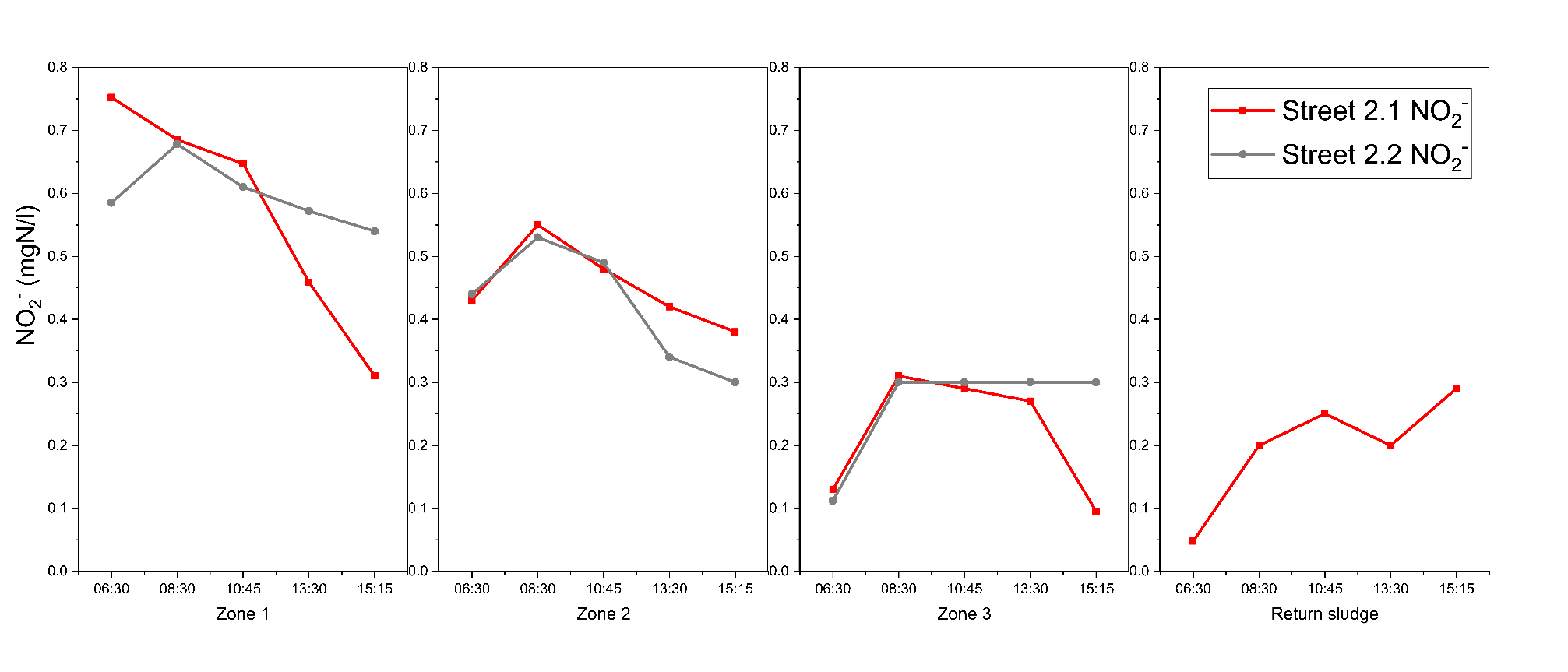

Figure S.7: Wastewater inflow on dry weather days (178 days between 01.11.19 and 27.02.21)
